# DenseFusion-DA2: End-to-End Pose-Estimation Network Based on RGB-D Sensors and Multi-Channel Attention Mechanisms

**DOI:** 10.3390/s24206643

**Published:** 2024-10-15

**Authors:** Hanqi Li, Guoyang Wan, Xuna Li, Chengwen Wang, Hong Zhang, Bingyou Liu

**Affiliations:** Department of Electrical Engineering, Anhui Polytechnic University, Beijing Road No. 8, Wuhu 241000, China; 18856621799@163.com (H.L.); 17355386718@163.com (X.L.); 18255354748@163.com (C.W.); zhang666y@126.com (H.Z.); lby009@mail.ustc.edu.cn (B.L.)

**Keywords:** pose estimation, attention mechanism, deep learning, CNN, feature fusion

## Abstract

Notably, 6D pose estimation is a critical technology that enables robotics to perceive and interact with their operational environment. However, occlusion causes a loss of local features, which, in turn, restricts the estimation accuracy. To address these challenges, this paper proposes an end-to-end pose-estimation network based on a multi-channel attention mechanism, DA2Net. Firstly, a multi-channel attention mechanism, designated as “DA2Net”, was devised using A^2^-Nets as its foundation. This mechanism is constructed in two steps. In the first step, the essential characteristics are extracted from the global feature space through the second-order attention pool. In the second step, a feature map is generated by the integration of position and channel attention. Subsequently, the extracted key features are assigned to each position of the feature map, enhancing both the feature representation capacity and the overall performance. Secondly, the designed attention mechanism is introduced into both the feature fusion and pose iterative refinement networks to enhance the network’s capacity to acquire local features thus improving its overall performance. The experimental results demonstrated that the estimation accuracy of DenseFusion-DA2 on the LineMOD dataset was approximately 3.4% higher than that of DenseFusion. Furthermore, the estimation accuracy surpassed that of PoseCNN, PVNet, SSD6D, and PointFusion by 8.3%, 11.1%, 20.3%, and 23.8%, respectively. The estimation accuracy also shows a significant advantage on the Occluded LineMOD and HR-Vision datasets. This research not only presents a more efficient solution for robot perception but also introduces novel ideas and methods for technological advancements and applications in related fields.

## 1. Introduction

Object 6DOF pose estimation refers to determining the six-degrees-of-freedom (6DOF) pose of an object and is a key task in computer vision and robotics, i.e., determining the position (x, y, z) of an object in three-dimensional space, along with its 3D translation and rotation relative to a sensor (typically an RGB-D camera or other 3D imaging device). This technique is widely applied in robotic grasping and manipulation [1,2], autonomous driving [3,4,5], augmented reality (AR) [6], and virtual reality (VR) [7]. Ideally, the technique should exhibit robustness to severe occlusion, illumination variations, and background complexity. However, existing methods struggle to meet the demands of practical applications, requiring ongoing research and exploration.

Mainstream approaches to 6D pose estimation encompass both traditional and deep-learning methods. Traditional algorithms primarily rely on geometric and feature matching, extracting features such as points, lines, and circles from the target object and calculating its position and orientation using vision algorithms. These methods depend on feature data from sensors, typically RGB cameras and 3D laser scanners. Established algorithms include ICP [8], SIFT [9], SURF [10], ORB [11], EPnP [12], and PPF [13]. However, these algorithms rely heavily on manually designed features and geometric models for tasks such as point-cloud alignment, feature point extraction, or establishing correspondences between 2D and 3D feature points, making them inefficient in complex environments or with large-scale data and thus hindering effective pose estimation.

In recent years, with the emergence of advanced technologies like deep learning and convolutional neural networks (CNNs), deep learning-based algorithms have gained significant attention. Many researchers have applied these techniques to 6D pose estimation, and these methods can automatically learn high-level features from sensor data through large-scale data training, thereby achieving more accurate and robust pose estimation in complex environments. Deep-learning methods for pose estimation can be classified into the following three categories based on the type of input data: RGB images, RGB-D images, and point clouds. Methods using RGB images rely solely on visible light captured by sensors, characterized by low acquisition cost and minimal equipment requirements, but they struggle with illumination variations and occlusions. The point-cloud approach, in contrast, directly captures 3D point-cloud data through sensors (e.g., LIDAR or structured light cameras), providing rich spatial information but entailing high data acquisition costs and processing complexity, despite achieving high accuracy. The RGB-D image approach combines information from both RGB and depth images (usually obtained from depth cameras such as Kinect or RealSense) and, by leveraging both data types, it exhibits stronger robustness against illumination changes and occlusions, enabling more accurate object pose estimation.

Although significant progress has been made in many aspects of pose-estimation algorithms based on RGB-D images, significant challenges remain in addressing heavily occluded or complex environments. One paper [14] has highlighted that significant light variations and heavy occlusions present major challenges in the grasping operations of home robots for known household objects. To further enhance the performance of the RGB-D pose-estimation algorithm in such practical applications and improve estimation accuracy, this paper proposes an end-to-end pose-estimation algorithm based on the multi-channel attention mechanism, DA2Net. The main contributions of this study are as follows:

(1) To better address the aforementioned challenges, prior to enhancement, a multi-channel attention mechanism, DA2Net, was designed based on A^2^-Nets [15], incorporating both a position attention module and a channel attention module. This mechanism excels in associating features and redistributing global information while simultaneously improving the model’s ability to capture both local and global contextual information. This capability is key to the superior performance of DenseFusion-DA2 proposed in this paper.

(2) With DenseFusion [16] serving as the primary framework, DA2Net was integrated into the feature-fusion network and the iterative refinement network to enhance the understanding of local details and infer occluded parts from global information.

(3) To comprehensively evaluate the performance of the improved network in practical scenarios, a custom LineMOD dataset named HR-Vision was developed to simulate the arrangement of household items and their placement environments under varying lighting intensities, addressing the challenges faced by domestic robots in grasping tasks. Experimental results indicate that DenseFusion-DA2 achieves a 3.4% improvement in estimation accuracy over DenseFusion on the public LineMOD dataset. On the challenging Occluded LineMOD and HR-Vision datasets, the improvements are 3% and 8.3%, respectively.

## 2. Related Work

RGB image methods: These methods primarily rely on visible light images for pose estimation and are widely employed across various application scenarios due to the low cost of image acquisition and minimal equipment requirements. However, they often face greater challenges when dealing with illumination variations, occlusions, and complex backgrounds. For instance, OpenPose [17], which employs a multi-stage convolutional neural network to extract human body keypoints from RGB images, is a pioneering contribution to this field. AlphaPose [18] subsequently improved detection precision and robustness through multi-task learning and a top–down framework. Recent approaches, such as HRNet [19], have introduced high-resolution network structures to fuse features at multiple scales, further improving pose estimation accuracy. Despite these advancements, 3D pose estimation continues to be a major challenge due to the lack of depth information.

Point-cloud methods: These methods employ three-dimensional point-cloud data for pose estimation, providing comprehensive spatial information and highly accurate 3D pose estimations. However, the acquisition of point-cloud data is expensive, and its processing is complex, involving significant computational and storage challenges. Methods like PointNet [20] and PointNet++ [21] are state-of-the-art in point-cloud processing, offering efficient feature extraction directly from point-cloud data. The dynamic graph convolutional network (DGCNN) [22] further improves feature representation capabilities. More recently, Point Transformer [23] employed the Transformer [24] architecture to process point-cloud data, achieving leading performance across various tasks. Although these methods efficiently encode geometric information from point clouds, they are often costly.

RGB-D image methods: Methods integrating data from both RGB and depth images allow for more precise 3D pose estimation by fusing the two data types. Compared with single RGB images or point clouds, RGB-D methods capture a more comprehensive range of environmental information, enhancing the robustness and accuracy of pose estimation. DenseFusion is a leading approach in RGB-D pose-estimation algorithms, where the fusion of RGB and depth features enables accurate 6D pose estimation. PoseCNN [25] estimates object poses using RGB-D data through segmentation and regression, while G2L-Net [26] optimizes the RGB-D fusion strategy and feature-extraction network, achieving excellent results on multiple datasets. However, these methods are overly dependent on post-processing steps to fully capture 3D information. Recently, many advanced algorithms have been proposed to enhance the fusion of RGB and depth features. For example, FFB6D [27] introduces a fully streamed bidirectional fusion network, incorporating a bidirectional fusion module to facilitate information exchange across each encoding and decoding layer. While it performs well on specific datasets, such as YCB-Video and LineMOD, its performance is constrained on datasets characterized by more severe occlusions and drastic variations in lighting. Geometry Attention Fusion [28] employs a color and geometry attention mechanism to improve the model’s understanding of object shape and surface information, but its effectiveness is reduced when handling highly occluded objects. The dataset used in this approach estimates the pose of a single object, without addressing occlusions. CMA [29] introduces a cross-modal attention mechanism that adaptively combines RGB and depth features using attention weights. However, it relies heavily on convolution operations, leading to the loss of detailed features, which affects its performance when handling objects with complex or irregular shapes. To enhance its applicability for domestic robot grasping and address specific challenges, this paper designs and introduces the multi-channel attention module DA2Net, based on DenseFusion [16], to overcome these limitations.

Results demonstrated that the enhanced DenseFusion-DA2 network improved performance compared with the original DenseFusion [16]. DenseFusion-DA2 demonstrated enhanced robustness in handling light variations and occlusion issues, with an estimated accuracy improvement of 3.4% on the LineMOD dataset compared with DenseFusion [16]. Furthermore, it outperformed PointFusion [30], PVNet [31], SSD6D [32], and PoseCNN [25]. In addition, the estimation accuracy also shows a significant advantage on the Occluded LineMOD and HR-Vision datasets.

## 3. Methodology

Addressing the limitations of existing deep learning-based pose-estimation algorithms, this paper presents the design of a multi-channel attention mechanism, DA2Net, based on A^2^-Nets [15], which is integrated into DenseFusion [16] to improve both the feature fusion and iterative refinement networks. This chapter comprises the following four main sections: (1) Method Overview, (2) Multi-channel Attention Mechanism DA2Net, (3) Improved Feature-Fusion Network, and (4) Improved Iterative Refinement Network DA2iter.

### 3.1. Method Overview

**Figure 1:**
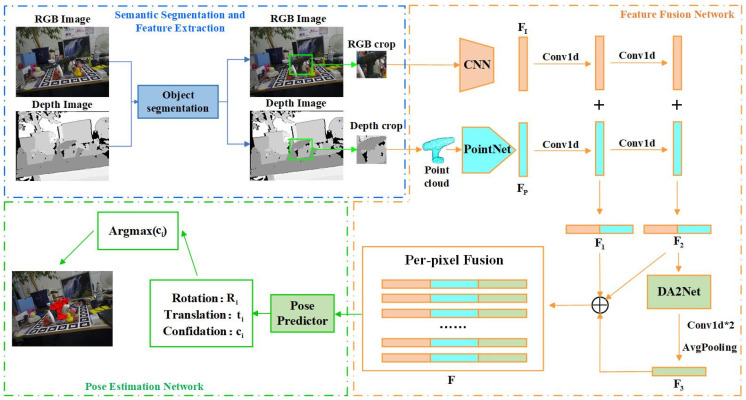
DenseFusion-DA2 network architecture (the * in the figure is the multiplication sign).

The DenseFusion-DA2 network, as developed in this paper, can be divided into the following three key segments (as shown in Figure 1): (1) semantic segmentation and feature extraction, (2) feature-fusion network, and (3) pose-estimation network.

Semantic Segmentation and Feature Extraction: The segmentation framework employed here uses an encoder translator architecture within PoseCNN [25]. For the input RGB-D image, the color map $I\in R^{H\times W\times 3}$ and depth map of the target object are cropped from the input RGB and depth images, respectively, using semantic segmentation. The depth map is then converted to a point cloud map $P\in R^{N\times 3}$ using the camera’s internal parameters. The color feature $F_{I}\in R^{N\times d_{rgb}}$ and the geometric feature $F_{P}\in R^{N\times 3}$ are then independently extracted using separate feature-extraction networks. Specifically, the color feature-extraction network uses an encoder decoder that employs ResNet18 [33] for the encoder and four upsampling layers (structured as PSPNet pyramids) for the decoder [34]. This process maps a color image of size H×W×3 into a space of size $H\times W\times d_{rgb}$, with each pixel represented by a $d_{rgb}$ dimensional vector corresponding to the color feature of the target object. The geometric feature-extraction network utilizes the PointNet [20] variant structure in DenseFusion [16] to extract geometric features from the point cloud of size N×3, where N denotes the number of point clouds (500 in this paper), yielding a geometric feature $F_{P}$ of size N×3.

Feature-Fusion Network: The core concept of this network is the layer-by-layer and pixel-by-pixel integration of features, aiming to make predictions based on the visible portions of the object. To facilitate the fusion of the two feature types, the color feature $F_{I}$ and the geometric feature $F_{P}$ are first subjected to convolution and activation functions, ensuring identical dimensions (color feature $F_{I1}{\in R}^{N\times d_{1}}$ and geometric feature $F_{P1}{\in R}^{N\times d_{1}}$). The two features are then passed through multilayer convolution and activation operations for pixel-by-pixel fusion, resulting in a fused feature $F{\in R}^{N\times \left(d_{1}+d_{2}+d_{3}\right)}$. This paper proposes an improved feature-fusion network based on the DA2Net attention mechanism, which enriches global and local contextual information during the multimodal feature-fusion process while minimizing the effects of occlusion and illumination changes. The improved network is discussed in subsequent sections.

Pose-Estimation Network: The fused pixel-level features F are then fed into a neural network for pose estimation, yielding the angular offset, translation parameters, and confidence level for each pixel, which are used to derive the object’s pose. This paper also incorporates an improved iterative refinement network based on DA2Net to further optimize pose estimation and enhance accuracy.

### 3.2. Multi-Channel Attention Mechanism DA2Net

#### 3.2.1. Attention Module

In the field of computer vision, traditional convolutional neural networks (CNNs) perform well in capturing local features but struggle to capture global features and long-range dependencies. To address this limitation, various attention mechanisms have been proposed, such as channel attention (SE-Net) [35] and spatial attention (non-local neural networks) [36]. However, these methods, which rely on channel or spatial attention alone, may overlook the relevance of other dimensions, thereby restricting the comprehensive utilization of multidimensional feature information. To overcome some of the limitations of existing mechanisms, attention mechanisms such as A^2^-Nets [15] and DANet [37] have been proposed.

**Figure 2:**
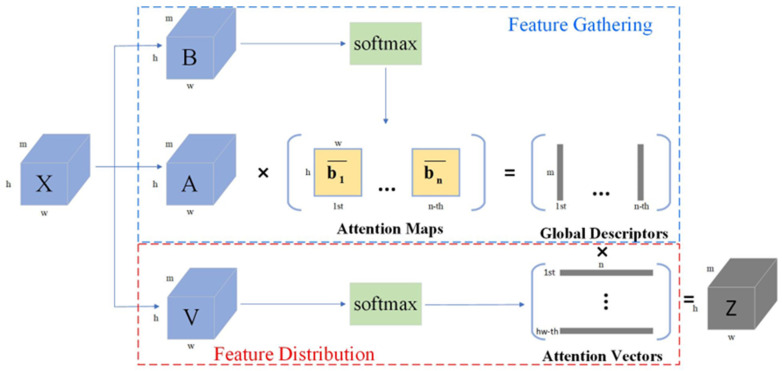
A^2^-Nets module.

A^2^-Nets: The A^2^-Nets (double attention networks) [15] attention mechanism offers a new approach to improving traditional attention mechanisms by better capturing the relationships and dependencies between different regions in an image, thereby improving feature representation and model performance. It achieves this through two branches, and its network structure is shown in Figure 2 as follows: the first, a feature-gathering branch, collects features from the entire space using a second-order attention pool, while the second, a feature-distribution branch, adaptively selects and allocates features from this collection to each location via another attention mechanism.

DANet: DANet (dual attention network) [37] is an advanced attention mechanism developed in recent years within the fields of deep learning and computer vision, designed to enhance feature representation and model performance. Its key innovation is the inclusion of a position attention module (PAM) and a channel attention module (CAM) to capture global dependencies between any two positions and any two channels.

#### 3.2.2. DA2Net

Local features are rich in detail but susceptible to occlusion, while global features provide a holistic representation of image information. Each has its own advantages. To efficiently leverage both local and global features, this paper builds upon A^2^-Nets [15] by retaining its dual-path structure and incorporating the channel attention module (CAM) and positional attention module (PAM) into the feature-allocation path, thereby proposing the DA2Net attention module. This allows for the capture of more complex global information using a compact set of features, enabling each position to receive customized global information. This complements the existing local features, facilitates the learning of complex relationships, captures intricate dependencies in the image, enhances detail understanding, and compensates for deficiencies in local feature extraction.

**Figure 3:**
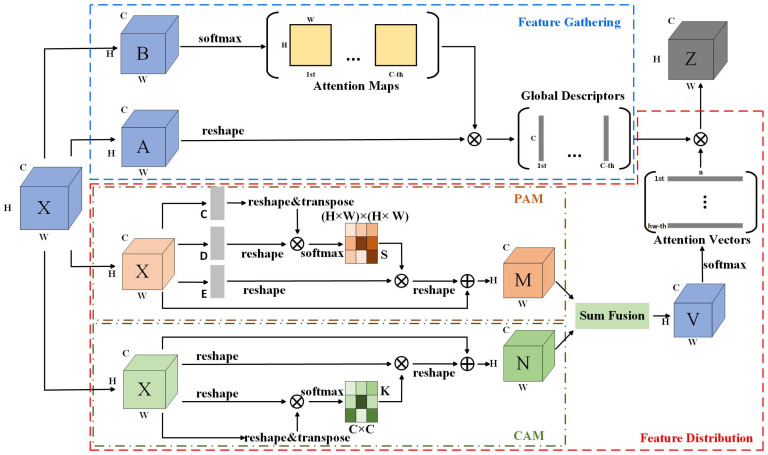
DA2Net module.

In the feature-fusion network of DenseFusion-DA2, color and geometric features, following a two-layer convolutional activation operation, are fused at the channel level to obtain the fused features $F_{2}\in R^{N\times d_{2}}$. An additional dimension is then added to generate $X\in R^{H\times W\times d_{2}}$. The DA2Net structure comprises two branches, namely the key feature-gathering branch and the multi-channel feature-distribution branch. The specific structure of DA2Net is illustrated in Figure 3. Both branches take reshaped fusion features X as input.

The key feature-gathering branch is designed to extract essential features from the input and collect them into a compact set, referred to as the “Global Descriptors.” First, X is reshaped into two feature maps $A=\left[a_{1},\cdots ,a_{HW}\right]\in R^{HW\times d_{2}}$ and $B=\left[b_{1},\cdots ,b_{HW}\right]\in R^{HW\times d_{2}}$. This operation reduces the dimensionality of the last dimension into the previous one, simplifying subsequent processing. The feature maps A and B are then subjected to a bilinear pooling operation, involving the outer product of the pair of feature vectors $\left(a_{i},b_{i}\right)$ from the two feature maps and their subsequent summation. The branch can be defined as follows:

(1) $$G_{gather}\left(X\right)=G_{bilinear}\left(A,B\right)=A\cdot softmax{\left(B\right)}^{T}$$

The output variable $G=\left[g_{1},\cdots ,g_{d_{2}}\right]\in R^{d_{2}\times d_{2}}$ is referred to as the set of global features.

Attention then shifts to the multi-channel feature-distribution branch. Before feature assignment, the positional attention module (PAM) and the channel attention module (CAM) are applied. The outputs from both attention modules are aggregated to obtain the feature map V. Finally, the features in the set G, produced by the upper branch, are assigned to V, and the final output is the feature map Z. Feature *X* is employed as the input to the two attention modules.

In the PAM module, the input feature X is initially passed through a convolutional layer and reshaped in order to obtain two new feature maps, $C{\in R}^{HW\times d_{2}}$ and $D{\in R}^{HW\times d_{2}}$, where N=H×W represents the number of pixels. The transpositions of C and D are then multiplied, generating the spatial attention map $S\in R^{HW\times HW}$ using the softmax function as follows:

(2) $$s_{ji}=\frac{exp\left(C_{i}\cdot D_{j}\right)}{\sum_{i=1}^{N}exp\left(C_{i}\cdot D_{j}\right)}$$

where $s_{ji}$ represents the effect of the $i^{th}$ position on the $j^{th}$ position. Another feature map, E, is obtained by passing X through another convolutional layer and reshaping it to $E\in R^{HW\times d_{2}}$. The matrix E is multiplied by the transpose of S, scaled by a parameter α, and then combined with X through weighted summation to obtain the final result M:

(3) $$M_{j}=\alpha \sum_{i=1}^{N}\left(s_{ji}E_{i}\right)+X_{j}$$

where α is initialized at 0 and undergoes a gradual learning process, whereby its weight is increased over time. In the CAM module, the channel attention map $K\in R^{d_{2}\times d_{2}}$ is directly computed from the input features X as follows:

(4) $$k_{ji}=\frac{exp\left(X_{i}\cdot X_{j}\right)}{\sum_{i=1}^{d_{2}}exp\left(X_{i}\cdot X_{j}\right)}$$

where $k_{ji}$ represents the influence of the $i^{th}$ channel on the $j^{th}$ channel. The transpose of K is then multiplied with X, reshaped to $R^{d_{2}\times H\times W}$, then multiplied by a scaling factor β and combined with X through weighted summation to obtain the final result $N\in R^{d_{2}\times H\times W}$ as follows:

(5) $$N_{j}=\beta \sum_{i=1}^{d_{2}}\left(k_{ji}X_{i}\right)+X_{j}$$

where β is set to 0, and it is subsequently adjusted to achieve larger weights through a process of learning. Finally, the outputs of the two modules are element-wise summed to complete feature fusion and reshaped into the feature map $V\in R^{HW\times d_{2}}$ as in the following equation:

(6) $$v_{i}=Sum\left(M_{j},N_{j}\right)$$

The combination of the two branches allocates the features from set G to each position in V, producing the final feature map Z of the module as follows:

(7) $$z_{i}=\sum_{\forall j}v_{ij}g_{j}=G_{gather}\left(X\right)v_{i}$$

In conclusion, the DA2 attention mechanism can be expressed by the following equation:

(8) $$\begin{array}{ll} Z & =F_{distr}\left(G_{gather}\left(X\right),V\right)\\ & =G_{gather}\left(X\right)Sum\left(M_{j},N_{j}\right)\\ & =A\cdot softmax{\left(B\right)}^{T}Sum(M_{j},N_{j}) \end{array}$$

where Sum is element-wise addition.

### 3.3. Improved Feature-Fusion Network

In this paper, an improved pixel-level feature-fusion network is designed to obtain pixel-point features for subsequent bit-position estimation by performing multilayer convolution and layer-by-layer fusion of two separate features. Due to occlusion problems, there may be occluded regions outside the convolution kernel’s receptive field or incomplete local information, causing the convolution operation to inadequately capture features in these regions, leading to local feature loss. To enhance the network’s ability to aggregate information, the DA2Net attention mechanism is introduced after the second layer of feature fusion. This design is illustrated in Figure 1, marked by the orange box on the right.

First, the color feature $F_{I}\in R^{N\times d_{rgb}}$ and the point-cloud feature $F_{P}\in R^{N\times 3}$ are input. After multilayer convolutional processing, the outputs of the first and second layers are fused through channel fusion, generating two distinct fusion features, $F_{1}{\in R}^{N\times d_{1}}$ and $F_{2}{\in R}^{N\times d_{2}}$. Subsequently, the fusion feature $F_{2}$ is input into the DA2Net module to capture local and global contextual information, assigning global features to each position on the feature map through position and channel attention, thereby comprehensively acquiring diverse feature information from the image. This process is designed to efficiently recognize and process features within the occluded regions. After applying two layers of convolution and average pooling to the features enhanced by the DA2Net module, a new fusion feature $F_{3}\in R^{N\times d_{3}}$ is generated. Finally, $F_{1}$, $F_{2}$, and $F_{3}$ are concatenated along the channel dimension, forming a new pixel-level fusion feature $F\in R^{N\times \left(d_{1}+d_{2}+d_{3}\right)}$.

### 3.4. Improved Iterative Refinement Network DA2iter

**Figure 4:**
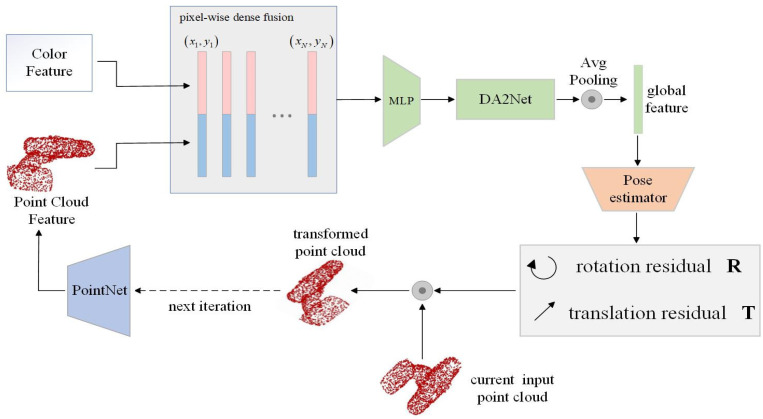
Iterative refinement of the network processing flow.

Once the bit-pose estimation results are obtained, iterative refinement of the original network is enhanced using the DA2Net module to optimize the initially estimated poses. While CNN-based iterative refinement is employed, local feature loss remains an issue in the original refinement due to the use of multilayer convolution, limiting the effectiveness of bit-pose refinement. Therefore, before global feature extraction, the DA2Net attention mechanism is incorporated to address this issue, enabling rapid and robust pose refinement. The refinement process is shown in Figure 4.

In this iterative refinement network, the predicted pose from the previous iteration is used as part of the input for the next iteration. Additionally, the extracted color features from the main network are reused in each iteration to achieve a final 6D pose with high accuracy. After n iterations, the final pose estimated by the network is as follows:

(9) $$\left\{\begin{array}{c} T_{k+1}=T_{k}+∆T_{k}\\ T=T_{n}\times T_{n-1}\times \cdots \cdots \times T_{2}\times T_{1}\times T_{0} \end{array}\right.$$

where $T_{k}$ is the pose of the $k^{th}$ iteration, while $∆T_{k}\;$ is the predicted pose correction, T denotes the true pose of the object, and $T_{0}$ denotes the initial pose output by the pose-estimation network.

## 4. Experiments

In the experimental section, the objective was to verify whether the proposed DenseFusion-DA2 network could achieve strong performance in the presence of complex backgrounds, weak textures, and severe occlusion. Accordingly, the experiments were conducted on a computer equipped with an RTX A6000 GPU, training and testing the networks 200 times each. The training environment was built using PyTorch with the following hyperparameters: a learning rate of 0.0008, batch size of 8, refine_marge set to 0.011, and iteration set to 4. Several authoritative public datasets are available for pose estimation, including YCB-Video [38], Occlusion LineMOD [39], and LineMOD [40]. YCB-Video contains multiple objects with rich textures, although occlusion between objects is relatively low in most scenes, differing from the high levels of occlusion found in real-world settings. Therefore, this paper trains and tests the network on the benchmark datasets LineMOD, Occluded LineMOD, and HR-Vision:

(1) LineMOD dataset: Widely adopted by classical methods [41,42] and learning-based methods [27,28,29,32], it included 13 everyday objects across 15,780 groups. Each group contained corresponding RGB maps, depth maps, 3D models, and 6DOF bit-pose annotations, with 2373 groups used for training and 13,407 for testing. These objects varied in shape, size, and texture, with many being textureless or weakly textured. High environmental complexity and significant lighting variations further complicated bit-pose estimation.

(2) Occluded LineMOD dataset: This dataset is particularly suitable for object detection tasks in occluded environments. It was created by annotating each scene in the LineMOD dataset, with a focus on complex scenarios involving inter-object occlusion, significantly increasing the challenge of pose estimation.

(3) HR-Vision dataset: This dataset is specifically tailored to address the challenges encountered by domestic robot grasping in this study. A 40-s video of domestic objects arranged in a room was captured using a Realsense D435i camera. The camera’s 20-megapixel RGB sensor and 3D sensor are capable of providing resolutions up to 1280 × 720 at 30 frames per second, or 840 × 480 at 90 frames per second. In this study, a frame rate of 30 frames per second is employed, with the image data saved frame by frame. A total of 7050 groups were generated, each containing corresponding RGB images, depth maps, 3D models of the objects, and 6DOF pose labels. The group serial numbers, which are multiples of 5, are extracted as the training set, while the remaining groups constitute the test set. The dataset includes 1410 groups in the training set and 5640 groups in the test set. To realistically simulate the placement rules and environments of household objects, the following two modes were used: selecting four or five types of household items and placing them in different configurations under varying light intensities, with continuous variation of the shooting angle to capture the different levels of object occlusion. Occlusion rates of 30% to 60%, along with changes in lighting conditions, serve to verify the performance advantages of the proposed network in this practical application.

### 4.1. Loss Function

This study utilized the loss function originally defined in the DenseFusion [16] network. The goal was to minimize pose estimation loss for each pixel, defined as the distance between a point sampled from the target model’s ground-truth pose and the corresponding point on the same model transformed by the predicted pose. The loss was calculated as follows:

(10) $$L_{i}^{p}=\frac{1}{M}\sum_{j=1}^{M}\left\|\left(Rx_{j}+t\right)-\left({\bar{R}}_{i}x_{j}+{\bar{t}}_{i}\right)\right\|$$

The above loss function applies only to asymmetric objects. For symmetric objects, the following function is used:

(11) $$L_{i}^{p}=\frac{1}{M}\sum_{j=1}^{M}\begin{array}{c} min\\ 0<n<M \end{array}\left\|\left(Rx_{j}+t\right)-\left({\bar{R}}_{i}x_{j}+{\bar{t}}_{i}\right)\right\|$$

where M denotes the number of sampling points, $x_{j}$ denotes the $j^{th}$ sampling point, $p=\left(R,t\right)$ is the true position of the object, and ${\bar{p}}_{i}=\left({\bar{R}}_{i},{\bar{t}}_{i}\right)$ denotes the predicted position generated by feature regression of the $i^{th}$ pixel point. The overall network loss function is defined as follows:

(12) $$L=\frac{1}{N}\sum_{i=1}^{N}\left(L_{i}^{p}c_{i}-wlog\left(c_{i}\right)\right)$$

where N is the total number of pixels, and w is a balancing hyperparameter. $c_{i}$ represents the confidence level associated with the pixel feature regression. In this study, the pose estimate with the highest confidence level was selected as the final output. This loss function has proven effective in the original network for pose estimation. To ensure a fair comparison with the original network’s performance, we retained the loss function in its original form.

### 4.2. Evaluation Indicators

To evaluate the accuracy of pose estimation, different metrics were used for the two types of objects. For asymmetric objects, the average distance of model points (ADD) was used as the evaluation metric. This measures the mean distance between the object vertices in the predicted pose ($R^{*}$, $t^{*}$) and the true pose (R, t), defined as follows:

(13) $$ADD=\frac{1}{m}\sum_{x\epsilon M}\left\|\left(Rx+t\right)-\left(R^{*}x+t^{*}\right)\right\|$$

where x represents a vertex of the object mesh o, and m is the total number of vertices. For symmetric objects, the average distance to the nearest point (ADD-S) was used. This metric calculates the average distance to the nearest point, as defined below:

(14) $$ADD-S\;=\frac{1}{m}\sum_{x_{1}\epsilon o}\begin{array}{c} min\\ x_{2}\in M \end{array}\left\|\left(Rx_{1}+t\right)-\left(R^{*}x_{2}+t^{*}\right)\right\|$$

### 4.3. Results

#### 4.3.1. Results on the LineMOD Dataset

(1) Contrast experiment

The performance evaluation results of the DenseFusion-DA2 model, as designed in this paper, on the LineMOD dataset using ADD(-S) as the evaluation metric are presented in Table 1.

**Figure 5:**
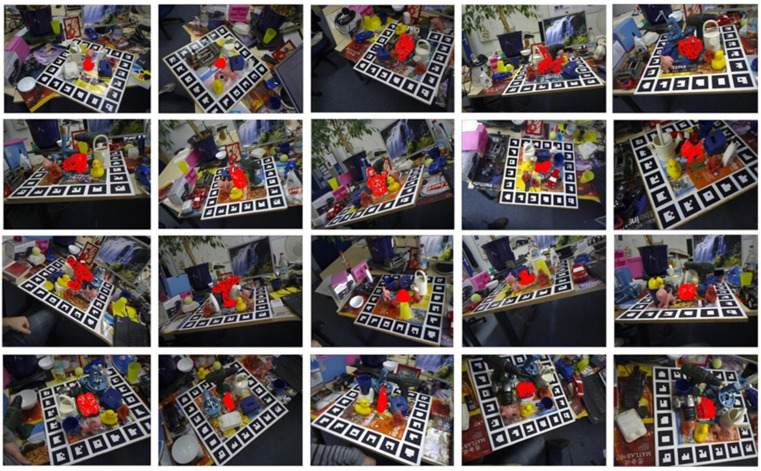
DenseFusion-DA2 pose-estimation visualization results on the LineMOD dataset.

The results in Table 1 show that DenseFusion-DA2 achieved an optimal accuracy of 96.7% on the LineMOD dataset, which was a 3.4% improvement over DenseFusion [16]. Additionally, comparisons with PoseCNN [25], PVNet [31], SSD6D [32], and PointFusion [30] showed that our approach improved accuracy by 8.3%, 11.1%, 20.3%, and 23.8%, respectively. These results show that under textureless and low-texture conditions, our method outperforms the above approaches, although FFB6D [27] achieves superior performance compared to our method. However, the objects in the LineMOD dataset are independent, non-overlapping, and clearly visible in the images with minimal occlusion. Thus, the advantage of DenseFusion-DA2 cannot be fully demonstrated using this dataset alone. This study focuses on pose estimation in the presence of significant occlusion and substantial lighting variations, which will be validated through comparative experiments using the Occluded LineMOD and HR-Vision datasets. Figure 5 shows the 6D pose visualization after iterative refinement of DenseFusion-DA2 on the LineMOD dataset, with rotating and translating point-cloud models overlaid on RGB images.

(2) Ablation experiment

To evaluate the efficacy of the DenseFusion-DA2 model for pose estimation, ablation experiments were conducted on the LineMOD dataset. Table 2 shows that replacing the improved feature-fusion network with the original network reduced estimation accuracy from 96.7% to 94.8% compared with DenseFusion-DA2. Similarly, replacing DA2iter with the original iterative network further decreased accuracy from 96.7% to 94.1% compared with DenseFusion-DA2. These results demonstrate that both enhanced modules contributed to improved estimation accuracy.

Additionally, to evaluate the effectiveness of the novel DA2-Attention mechanism in enhancing pose-estimation performance, ablation experiments were conducted on the LineMOD dataset. To ensure fairness, the improved iterative refinement network DAiter was used across all cases. Table 3 shows that introducing A^2^-Net [15] alone into the feature-fusion network decreased accuracy from 96.7% to 95.4% compared with the novel DA2Net mechanism designed in this paper. Similarly, using only DANet [37] resulted in a decrease in accuracy from 96.7% to 94.4%, demonstrating that the novel DA2Net mechanism offers significant advantages in capturing global and local feature context. This advantage leads to improved performance in the pose-estimation algorithm.

#### 4.3.2. Results on the Occlusion LineMOD Dataset

To verify the effectiveness of DenseFusion-DA2 in addressing severe occlusion challenges, a comparative experiment was performed using the Occlusion LineMOD dataset. The comparative results, evaluated using the ADD(-S) metric, are presented in Table 4. This dataset is an occluded version of the LineMOD dataset, which emphasizes object occlusion in complex scenes.

As shown in the table, the proposed network improves estimation accuracy by 18.2%, 6.4%, and 3% compared to FFB6D [27], PoseCNN [25], and DenseFusion [16], respectively. This demonstrates the robustness of the proposed method in handling occlusions.

#### 4.3.3. Results on the HR-Vision Dataset

To further validate the robustness of DenseFusion-DA2 against lighting variations and severe occlusions in achieving accurate pose estimation, comparative experiments were performed on the HR-Vision dataset, which presents more complex lighting conditions and more severe occlusions compared to the publicly available LineMOD and Occlusion LineMOD datasets. The results of comparing DenseFusion-DA2, DenseFusion [16], and FFB6D [27] on this custom dataset, based on the ADD(-S) metric, are shown in Table 5.

**Figure 6:**
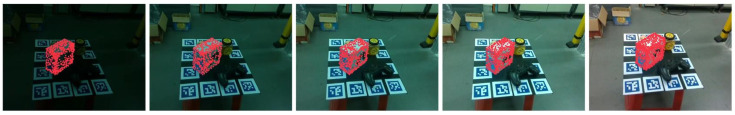
Visualization results of DenseFusion-DA2 pose estimation under different light intensities.

**Figure 7:**
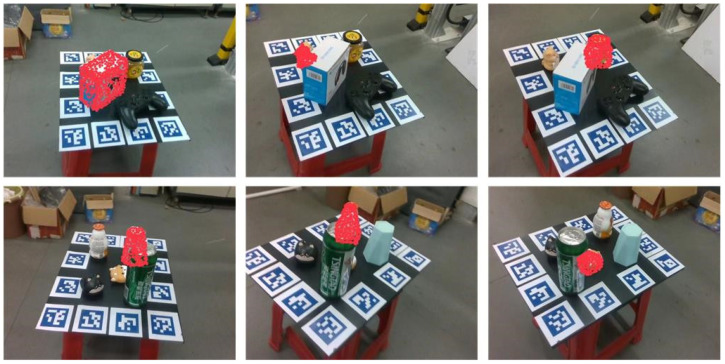
DenseFusion-DA2 pose-estimation visualization results on HR-Vision dataset.

As shown in the Table 5, the proposed network improves estimation accuracy on this dataset by 17.9% and 8.3%. This further demonstrates that the DenseFusion-DA2 network maintains effective pose estimation even under severe occlusions and lighting variations and confirms the advantages of this method in alignment with our research focus. Figure 6 illustrates the network’s performance in pose estimation under varying light intensities. Figure 7 shows the network’s visualization results on the custom LineMOD dataset, further demonstrating its effectiveness in handling severe occlusion and light changes.

## 5. Conclusions

To address local feature loss due to severe occlusion and significant illumination changes and to achieve accurate 6D pose estimation, this paper proposes an end-to-end pose-estimation algorithm, DenseFusion-DA2, based on the multi-channel attention mechanism DA2Net. The integration of the position attention module (PAM) and channel attention module (CAM) into A^2^-Nets [15] aimed at optimizing the feature allocation branch, resulted in the development of the new attention mechanism DA2Net. This mechanism was incorporated into both the feature-fusion network and iterative refinement network of DenseFusion [16], leading to significant performance improvements. Experimental results showed that DenseFusion-DA2 exhibited greater robustness to illumination variations and occlusion, achieving an estimated accuracy improvement of approximately 3.4% compared with DenseFusion [16] on the LineMOD dataset. Furthermore, it outperformed PointFusion [30], PVNet [31], SSD6D [32], PoseCNN [25], and other deep-learning-based pose-estimation algorithms that use RGB-D images for 6D pose estimation. In particular, the estimation accuracy exhibited significant advantages on the Occluded LineMOD and HR-Vision datasets with complex backgrounds, which aligns with the focus of our research. This verifies the advantages of DenseFusion-DA2 in pose estimation for the practical application of grasping objects by domestic robots. In conclusion, our method introduces a novel approach for capturing both local and global features and effectively addressing 6D pose estimation challenges under significant occlusion and illumination variations.

## Figures and Tables

**Table 1:** Performance comparison of the algorithms on the LineMOD dataset (The most accurate values are bolded).

	PointFusion [30]	SSD6D [32] _ICP	PVNet [31]	PoseCNN [25]	DenseFusion [16]	FFB6D [27]	Ours
ape	71.8	64.8	42.9	76.4	85.9	**97.6**	90.3
ben.	78.5	78.9	**99.5**	97.0	93.6	99.3	98.5
cam	61.0	78.0	85.7	93.5	91.1	**99.0**	96.2
can	59.7	85.7	95.5	96.5	95.2	**98.7**	97.1
cat	77.3	70.2	79.5	82.1	95.3	**99.2**	94.8
drill.	50.2	72.5	94.1	95.3	90.0	**98.5**	96.5
duck	63.1	66.4	50.8	78.0	87.5	97.8	**98.1**
egg	97.4	**100.0**	99.7	97.1	99.8	100.0	99.5
glue	97.9	100.0	94.9	99.2	99.8	**100.0**	**100.0**
hole.	70.0	48.9	81.7	52.8	90.3	**98.7**	95.0
iron	84.8	77.2	97.6	97.3	95.5	**98.4**	97.8
lamp	60.9	72.7	**98.3**	97.5	94.7	99.7	97.5
pho.	75.4	78.5	92.0	87.5	94.9	**99.7**	96.8
MEAN	72.9	76.4	85.6	88.4	93.3	**98.9**	96.7

**Table 2:** Ablation experiments of different improvement modules on the LineMOD dataset. (Ablation experiments were conducted to evaluate the different improved modules proposed in this paper, including the enhanced feature-fusion network and the iterative refinement network, using the LineMOD dataset and DenseFusion (DF) [16]. Here, ‘iter’ refers to the original iterative network, while ‘DA2iter’ denotes the improved iterative refinement network.

	Ape	Ben.	Cam	Can	Cat	Drill.	Duck	Egg	Glue	Hole.	Iron	Lamp	Pho.	MEAN
DF(iter) + DA2Net	86.3	94.8	90.2	95.2	**94.9**	93.8	90.4	99.2	99.5	92.1	95.9	95.5	96.0	94.1
DF(DA2iter)	88.0	94.2	95.6	95.9	94.1	93.1	88.3	**99.8**	99.5	93.9	**98.0**	96.0	**96.9**	94.8
Ours	**90.3**	**98.5**	**96.2**	**97.1**	94.8	**96.5**	**98.1**	99.5	**100**	**95.0**	97.8	**97.5**	96.8	**96.7**

**Table 3:** Ablation experiments on the LineMOD dataset using different attention mechanisms. Ablation experiments of feature-fusion networks with different attention mechanisms A^2^-Nets [15], DANet [37], and DA2Net on LineMOD dataset (all using improved iterative refinement).

	Ape	Ben.	Cam	Can	Cat	Drill.	Duck	Egg	Glue	Hole.	Iron	Lamp	Pho.	MEAN
DF + A^2^-Nets [15]	86.0	97.2	95.4	95.7	94.4	86.6	91.4	**99.8**	99.5	94.9	**98.5**	96.8	**97.0**	95.4
DF + DANet [37]	84.1	95.0	93.8	96.1	**95.9**	91.1	91.6	99.6	99.8	92.7	96.9	95.5	95.2	94.4
Ours	**90.3**	**98.5**	**96.2**	**97.1**	94.8	**96.5**	**98.1**	99.5	**100**	**95.0**	97.8	**97.5**	96.8	**96.7**

**Table 4:** Performance comparison of various algorithms on the Occluded LineMOD dataset.

	Ape	Can	Cat	Driller	Duck	Eggbox	Glue	Hole.	MEAN
FFB6D [27]	47.2	85.2	45.7	81.4	53.9	70.2	60.1	85.9	66.2
PoseCNN [25] + ICP	76.2	87.4	52.2	90.3	**77.7**	72.2	76.7	**91.4**	78.0
DenseFusion [16]	73.2	88.6	**72.2**	92.5	59.6	94.2	92.6	78.7	81.4
Ours	**82.8**	**90.5**	71.4	**93.2**	68.1	**95.5**	**94.7**	79.2	**84.4**

**Table 5:** Comparison of the performance of various algorithms on the HR-Vision dataset.

	Box	Toy	Glass Bottle	Cup	Headset	Milk	MEAN
FFB6D [27]	82.6	45.2	79.8	74.2	61.8	70.3	69.0
DF [16]	94.2	52.8	85.1	86.7	74.7	78.1	78.6
Ours	**98.3**	**70.6**	**89.2**	**90.8**	**85.3**	**87.0**	**86.9**

## Data Availability

Data are contained within the article.

## References

[1] Tremblay J., To T., Sundaralingam B., Xiang Y., Fox D., Birchfield S. (2018). Deep object pose estimation for semantic robotic grasping of household objects. arXiv.

[2] Bohg J., Morales A., Asfour T., Kragic D. (2013). Data-driven grasp synthesis—A survey. IEEE Trans. Robot..

[3] Chen X., Kundu K., Zhang Z., Ma H., Fidler S., Urtasun R. Monocular 3d object detection for autonomous driving. Proceedings of the IEEE Conference on Computer Vision and Pattern Recognition.

[4] Geiger A., Lenz P., Urtasun R. Are we ready for autonomous driving? the kitti vision benchmark suite. Proceedings of the 2012 IEEE Conference on Computer Vision and Pattern Recognition.

[5] You Y., Wang Y., Chao W.L., Garg D., Pleiss G., Hariharan B., Campbell M., Weinberger K.Q. (2019). Pseudo-lidar++: Accurate depth for 3d object detection in autonomous driving. arXiv.

[6] Marchand E., Uchiyama H., Spindler F. (2015). Pose estimation for augmented reality: A hands-on survey. IEEE Trans. Vis. Comput. Graph..

[7] Ababsa F., Mallem M. Robust camera pose estimation using 2d fiducials tracking for real-time augmented reality systems. Proceedings of the 2004 ACM SIGGRAPH International Conference on Virtual Reality Continuum and its Applications in Industry.

[8] Rusinkiewicz S., Levoy M. Efficient variants of the ICP algorithm. Proceedings of the Proceedings Third International Conference on 3-D Digital Imaging and Modeling.

[9] Ng P.C., Henikoff S. (2003). SIFT: Predicting amino acid changes that affect protein function. Nucleic Acids Res..

[10] Bay H., Tuytelaars T., Van Gool L. (2006). Surf: Speeded up robust features. Proceedings of the Computer Vision–ECCV 2006: 9th European Conference on Computer Vision.

[11] Rublee E., Rabaud V., Konolige K., Bradski G. ORB: An efficient alternative to SIFT or SURF. Proceedings of the 2011 International Conference on Computer Vision.

[12] Lepetit V., Moreno-Noguer F., Fua P. (2009). EP n P: An accurate O (n) solution to the P n P problem. Int. J. Comput. Vis..

[13] Yanagi T., Okamoto K., Takita S. (1996). Effects of blue, red, and blue/red lights of two different PPF levels on growth and morphogenesis of lettuce plants. Int. Symp. Plant Prod. Closed Ecosyst..

[14] Wei A.H., Chen B.Y. (2020). Robotic object recognition and grasping with a natural background. Health Inform. J..

[15] Chen Y., Kalantidis Y., Li J., Yan S., Feng J. (2018). A^2^-nets: Double attention networks. Adv. Neural Inf. Process. Syst..

[16] Wang C., Xu D., Zhu Y., Martín-Martín R., Lu C., Fei-Fei L., Savarese S. Densefusion: 6d object pose estimation by iterative dense fusion. Proceedings of the IEEE/CVF Conference on Computer Vision and Pattern Recognition.

[17] Qiao S., Wang Y., Li J. Real-time human gesture grading based on OpenPose. Proceedings of the 2017 10th International Congress on Image and Signal Processing, BioMedical Engineering and Informatics (CISP-BMEI).

[18] Fang H.S., Li J., Tang H., Xu C., Zhu H., Xiu Y., Li Y.-L., Lu C. (2022). Alphapose: Whole-body regional multi-person pose estimation and tracking in real-time. IEEE Trans. Pattern Anal. Mach. Intell..

[19] Wang J., Sun K., Cheng T., Jiang B., Deng C., Zhao Y., Liu D., Mu Y., Tan M., Wang X. (2020). Deep high-resolution representation learning for visual recognition. IEEE Trans. Pattern Anal. Mach. Intell..

[20] Qi C.R., Su H., Mo K., Guibas L.J. Pointnet: Deep learning on point sets for 3d classification and segmentation. Proceedings of the IEEE Conference on Computer Vision and Pattern Recognition.

[21] Qi C.R., Yi L., Su H., Guibas L.J. (2017). Pointnet++: Deep hierarchical feature learning on point sets in a metric space. Adv. Neural Inf. Process. Syst..

[22] Diao Z., Wang X., Zhang D., Liu Y., Xie K., He S. (2019). Dynamic spatial-temporal graph convolutional neural networks for traffic forecasting. Proc. AAAI Conf. Artif. Intell..

[23] Zhao H., Jiang L., Jia J., Torr P.H., Koltun V. Point transformer. Proceedings of the IEEE/CVF International Conference on Computer Vision.

[24] Han K., Xiao A., Wu E., Guo J., Xu C., Wang Y. (2021). Transformer in transformer. Adv. Neural Inf. Process. Syst..

[25] Xiang Y., Schmidt T., Narayanan V., Fox D. (2017). Posecnn: A convolutional neural network for 6d object pose estimation in cluttered scenes. arXiv.

[26] Chen W., Jia X., Chang H.J., Duan J., Leonardis A. G2l-net: Global to local network for real-time 6d pose estimation with embedding vector features. Proceedings of the IEEE/CVF Conference on Computer Vision and Pattern Recognition.

[27] He Y., Huang H., Fan H., Chen Q., Sun J. Ffb6d: A full flow bidirectional fusion network for 6d pose estimation. Proceedings of the IEEE/CVF Conference on Computer Vision and Pattern Recognition.

[28] Yuan H., Veltkamp R.C. 6D object pose estimation with color/geometry attention fusion. Proceedings of the 2020 16th International Conference on Control, Automation, Robotics and Vision (ICARCV).

[29] Zou L., Huang Z., Wang F., Yang Z., Wang G. (2021). CMA: Cross-modal attention for 6D object pose estimation. Comput. Graph..

[30] Xu D., Anguelov D., Jain A. Pointfusion: Deep sensor fusion for 3d bounding box estimation. Proceedings of the IEEE Conference on Computer Vision and Pattern Recognition.

[31] Peng S., Liu Y., Huang Q., Zhou X., Bao H. (2019). PVNet: Pixel-wise voting network for 6DoF object pose estimation. IEEE Trans. Pattern Anal. Mach. Intell..

[32] Kehl W., Manhardt F., Tombari F., Ilic S., Navab N. Ssd-6d: Making rgb-based 3d detection and 6d pose estimation great again. Proceedings of the IEEE International Conference on Computer Vision.

[33] He K., Zhang X., Ren S., Sun J. Deep residual learning for image recognition. Proceedings of the IEEE Conference on Computer Vision and Pattern Recognition.

[34] Zhao H., Shi J., Qi X., Wang X., Jia J. Pyramid scene parsing network. Proceedings of the IEEE Conference on Computer Vision and Pattern Recognition.

[35] Hu J., Shen L., Sun G. Squeeze-and-excitation networks. Proceedings of the IEEE Conference on Computer Vision and Pattern Recognition.

[36] Wang X., Girshick R., Gupta A., He K. Non-local neural networks. Proceedings of the IEEE Conference on Computer Vision and Pattern Recognition.

[37] Fu J., Liu J., Tian H., Li Y., Bao Y., Fang Z., Lu H. Dual attention network for scene segmentation. Proceedings of the IEEE/CVF Conference on Computer Vision and Pattern Recognition.

[38] Calli B., Singh A., Walsman A., Srinivasa S., Abbeel P., Dollar A.M. The ycb object and model set: Towards common benchmarks for manipulation research. Proceedings of the 2015 International Conference on Advanced Robotics (ICAR).

[39] Brachmann E., Krull A., Michel F., Gumhold S., Shotton J., Rother C. (2014). Learning 6d object pose estimation using 3d object coordinates. Computer Vision–ECCV 2014: 13th European Conference, Zurich, Switzerland, 6–12 September 2014, Proceedings, Part II 13.

[40] Hinterstoisser S., Holzer S., Cagniart C., Ilic S., Konolige K., Navab N., Lepetit V. Multimodal templates for real-time detection of texture-less objects in heavily cluttered scenes. Proceedings of the 2011 International Conference on Computer Vision.

[41] Buch A.G., Kiforenko L., Kraft D. Rotational subgroup voting and pose clustering for robust 3d object recognition. Proceedings of the 2017 IEEE International Conference on Computer Vision (ICCV).

[42] Vidal J., Lin C.Y., Martí R. 6D pose estimation using an improved method based on point pair features. Proceedings of the 2018 4th International Conference on Control, Automation and Robotics (Iccar).

